# Tuberculosis of the Navicular Bone of the Left Foot Without Pulmonary Involvement in a Fifteen-Year-Old Indian Boy: A Rare Case Report

**DOI:** 10.7759/cureus.39930

**Published:** 2023-06-04

**Authors:** Sankalp Yadav, Gautam Rawal, Madhan Jeyaraman

**Affiliations:** 1 Internal Medicine, Shri Madan Lal Khurana Chest Clinic, Moti Nagar, New Delhi, IND; 2 Respiratory Medical Critical Care, Max Super Speciality Hospital, Saket, New Delhi, IND; 3 Orthopaedics, A.C.S. Medical College and Hospital, Dr. M.G.R. Educational and Research Institute, Chennai, IND

**Keywords:** culture and sensitivity, anti-tubercular therapy (att), navicular bone, bone tb, tuberculosis

## Abstract

Tuberculosis of the bones and joints is a relatively rare entity, even in endemic countries. The disease is an outcome of a *Mycobacterium tuberculosis* infection. Tuberculosis of the small bones of the foot is extremely rare, requires a high index of suspicion for establishing the diagnosis, and is often associated with delayed diagnosis, ultimately affecting the treatment outcomes.

Globally, tuberculosis of the navicular bone of the foot is an infrequently reported condition. We herein present a case of isolated tuberculosis of the navicular bone without pulmonary involvement. The patient reported complaints of pain and swelling in his left foot and underwent a detailed diagnostic workup. A final diagnosis was made with fine needle aspiration cytology, biopsy, culture, radiography, and magnetic resonance imaging (MRI). He was initiated on anti-tubercular chemotherapy for twelve months, with a substantial improvement in his symptoms. This case is very rare, as no such case with similar clinical features in this age group has ever been reported in the world.

## Introduction

Tuberculosis of the bones is rare [1]. It is usually seen in 1-3% of total tubercular patients [2]. It is an extrapulmonary manifestation of bacterial infection, and isolated cases without pulmonary involvement are seldom reported [3]. Nevertheless, it constitutes 15% of all extrapulmonary tuberculosis cases [3].

One such extremely rare presentation of bone involvement is tuberculosis of the bones of the foot [4]. Commonly involved bones of the foot are the navicular, calcaneum, talus, first metatarsal, and medial and intermediate cuneiforms [4]. Isolated tuberculosis of the navicular bone of the foot is extremely rare [1]. It is a difficult clinical condition to diagnose, and often these patients receive delayed care [1].

Herein, we present a case of isolated navicular bone tuberculosis in a 15-year-old Indian boy without any pulmonary involvement who presented with pain and swelling in his left foot. He was diagnosed after fine needle aspiration cytology of the samples, biopsy, radiography, and magnetic resonance imaging. Medical management with antitubercular chemotherapy for a total of twelve months was advised per the national guidelines, leading to the relief of symptoms [5]. To the best knowledge of the authors, this is the first such case in this age group ever reported in the world.

## Case presentation

A 15-year-old Indian male student belonging to low socioeconomic status came to the outpatient department with chief complaints of pain and swelling in his left foot for three months. The swelling was insidious in onset and has increased over the medial aspect of the dorsum of the left foot. It was associated with pain that was localized over the entire left foot. His pain was aggravated while walking and was relieved a little after taking over-the-counter nonsteroidal anti-inflammatory drugs (NSAIDs). He was incapable of bearing weight on the left foot, though there was no limp.

There was no history of fever, cough, night sweats, or weight loss. And there was no history of trauma. Besides, there was no history of tuberculosis among his close contacts. Further, there was no history of any major medical or surgical intervention in the past. He was fully vaccinated for his age.

A general examination was suggestive of a young child with a temperature of 98.4 degrees Fahrenheit, a pulse of 76 per minute, a blood pressure of 110/80 mmHg, a respiratory rate of 18 per minute, and oxygen saturation (SpO2) of 98 percent in room air.

**Figure 1:**
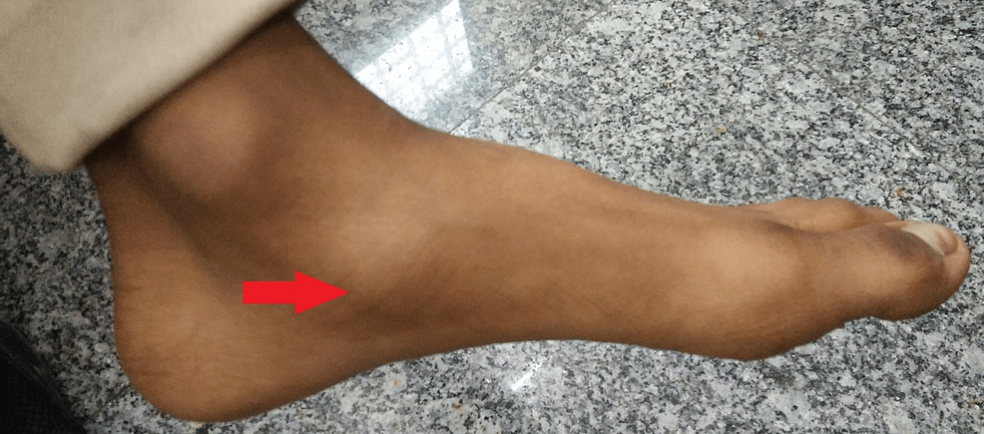
Swelling over the anteromedial surface of the dorsum of the left foot

The local examination was remarkable for a 4x3 cm swelling over the anteromedial surface of the dorsum of the left foot with a relatively smooth surface and tenderness on deep pressure over the navicular bone (Figure 1). The swelling was generalized and associated with a raised local temperature on the ipsilateral side of the overlying skin and did not subside on limb elevation. There were no visible ulcers, dilated veins, or discharging sinuses over it.

The eversion and inversion movements of the left foot were slightly limited and were associated with pain; however, dorsiflexion and plantar flexion were terminally painful, and still, a full range of movement of the foot was present. The right foot was normal. Further, there was no cyanosis, icterus, pallor, clubbing, koilonychia, or lymphadenopathy. The systemic examination was unremarkable.

Based on the presentation and clinical examination, a provisional diagnosis of pyogenic osteomyelitis of the foot was made with differentials such as tuberculous osteomyelitis, fungal osteomyelitis, bone tumors, and granulomatous diseases like gout or amyloidosis.

A detailed laboratory workup revealed an elevated erythrocyte sedimentation rate (ESR) of 50 mm during the first hour with normal blood counts. The C-reactive protein value was 7 mg/l. A rheumatoid factor test was negative, and his HIV was non-reactive. Induced sputum for the acid fast bacilli test, cartridge-based nucleic acid amplification test, and culture were negative.

**Figure 2:**
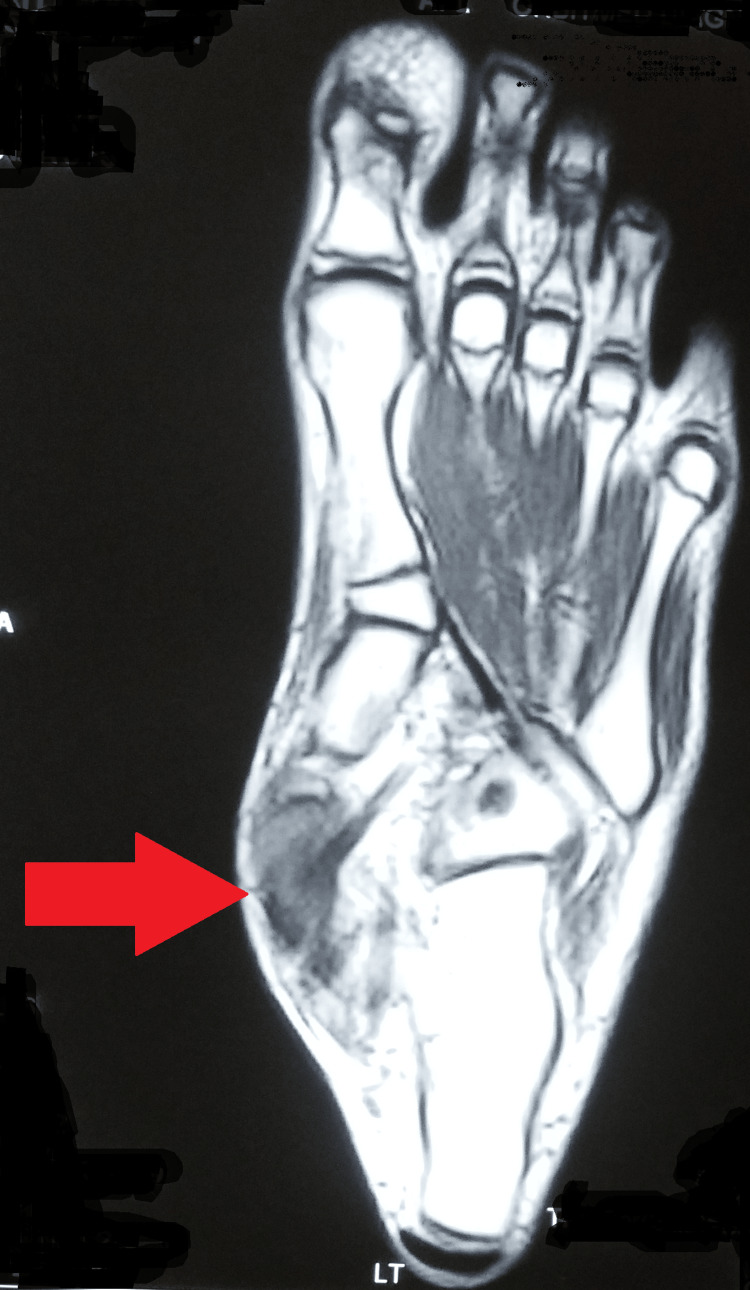
Magnetic resonance imaging of the left foot suggestive of focal erosion (proximal cortex) of the medial navicular bone with associated significant soft tissue edema and bone marrow edema

**Figure 3:**
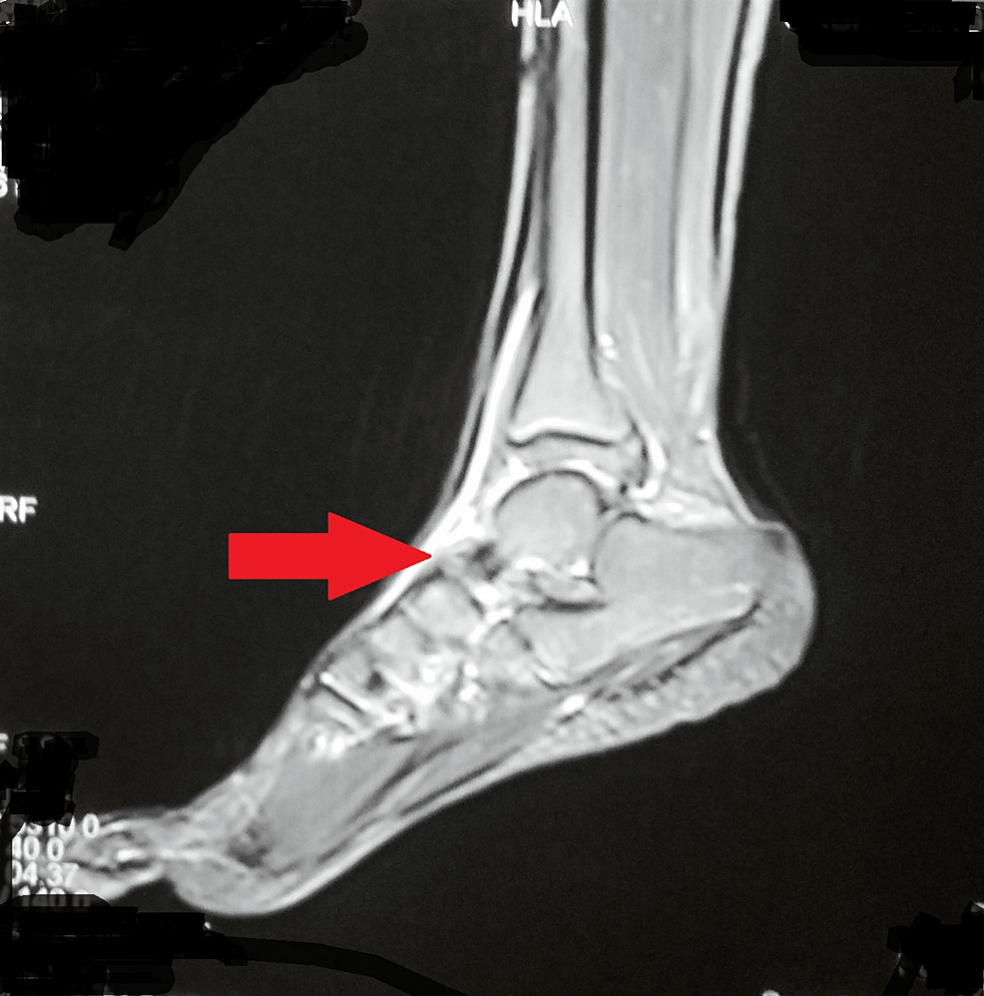
Magnetic resonance imaging of the left foot suggestive of focal erosion of the medial navicular bone

The plain radiograph of the left foot showed erosion of the navicular bone. A chest radiograph was within normal limits. Further, the magnetic resonance imaging (MRI) of the left foot was suggestive of focal erosion (proximal cortex) of the medial navicular bone with associated significant soft tissue edema and bone marrow edema (Figure 2 and Figure 3).

Aspiration cytology of the swelling was undertaken, and the aspirated fluid was found positive for acid-fast bacilli on Ziehl-Neelsen staining. Histopathology of the biopsy sample was remarkable for tuberculosis with epitheloid granulomas and caseating necrosis. Aspirated fluid was also sent for the cartridge-based nucleic acid amplification test, which revealed *Mycobacterium tuberculosis* detection (low) with no resistance to rifampicin. Another sample was sent to the National Reference Laboratory for line probe assay (LPA) and culture, and the results were suggestive of *Mycobacterium tuberculosis* being detected on LPA with no resistance to rifampicin or isoniazid and growing on the liquid culture system BACTEC (Becton, Dickinson and Company, Franklin Lakes, New Jersey, United States) on the eighteenth day with no resistance to the first-line anti-tubercular drugs. The joint fluid test from the talonavicular joint was not suggestive of urate crystals.

Finally, a diagnosis of primary navicular bone tuberculosis without pulmonary involvement was made, and he was initiated on anti-tubercular treatment per his weight for a total of 12 months, initially with four drugs for two months and followed with three anti-tubercular drugs for a period of 10 months, per the national guidelines (Table 1) [5].

**Table 1:** Details of anti-tubercular chemotherapy

Intensive Phase		
Drug	Dose	Duration
Rifampicin	450 mg	8 weeks
Pyrazinamide	1000 mg	8 weeks
Ethambutol	600 mg	8 weeks
Isoniazid	300 mg	8 weeks
Continuation Phase		
Rifampicin	450 mg	40 weeks
Ethambutol	600 mg	40 weeks
Isoniazid	300 mg	40 weeks

Along with this, he was given a tablet of pyridoxine 40 mg for the entire duration of 12 months and dietary advice for a high-protein diet. He fared well, with no major adverse drug reactions. Presently, he is on treatment and has completed seven months of treatment. His swelling has subsided, and there are no major complaints. His ESR has returned to normal limits, and on his request, he was transferred to his native village.

## Discussion

Extrapulmonary tuberculosis of the bones and joints, although rare, is increasingly reported [6]. Tuberculosis of the bones of the foot is difficult to diagnose due to atypical clinical features, the paucibacillary character of the disease, and a lack of awareness among the treating clinicians [1]. Often, there is a diagnostic and management delay [3,7]. This could adversely impact treatment outcomes [3,7].

There are various reports of tuberculosis of the navicular bones associated with other bones of the foot that are available in the literature, but isolated reports of navicular bone tuberculosis without pulmonary involvement in this age group are very rare [8].

A case similar to ours was presented by Boussetta et al. 2020 in a three-year-old child [8]. Our case shares similarities with theirs in the clinical features, site of the lesion, absence of constitutional symptoms of tuberculosis, and histopathological confirmation of the diagnosis [8]. However, our case differs from theirs in age, gender, absence of involvement of other bones and joints of the foot, and treatment duration [8]. Besides, unlike their case, there was no active curettage of navicular bone or talonavicular debridement done in our case [8].

Another case was reported by Kumar et al. in 2015 in a 47-year-old female [9]. Our case shares a few similarities with theirs. However, our case differs from theirs in age, gender, and the absence of any history of tuberculosis. Besides, there was no active abscess drain done in our case, which was repeated twice in their case [9].

Overall, the management of navicular bone tuberculosis is essentially medical, with antitubercular drugs [10]. However, surgical intervention is imperative in cases of failure of medical management or in cases of fistula, synovitis, or abscess [11]. For the correction of sequelae and painful deformities, arthrodesis surgery is indicated [11].

## Conclusions

Navicular bone tuberculosis is extremely rare. Isolated cases without any history of trauma or pulmonary involvement are challenging to diagnose. The present case would help the treating clinicians with this difficult-to-diagnose infection. Besides, early diagnosis is imperative in these cases to avoid unfavorable outcomes for patients.
